# The association of elevated reactive oxygen species levels from neutrophils with low-grade inflammation in the elderly

**DOI:** 10.1186/1742-4933-5-13

**Published:** 2008-10-24

**Authors:** Kishiko Ogawa, Katsuhiko Suzuki, Mitsuharu Okutsu, Kyoko Yamazaki, Shoji Shinkai

**Affiliations:** 1 Faculty of Human Sciences, Waseda University, 2-579-15 Mikajima, Tokorozawa, Saitama 359-1192, Japan; 2 Research Team for Social Participation and Health Promotion, Tokyo Metropolitan Institute of Gerontology, 35-2 Sakaecho, Itabashi, Tokyo 173-0015, Japan; 3 Faculty of Sports Sciences, Waseda University, 2-579-15 Mikajima, Tokorozawa, Saitama 359-1192, Japan; 4 Consolidated Research Institute for Advanced Science and Medical Care, Waseda University, 513 Wasedatsurumakicho Sinjuku, Tokyo 162-0041, Japan

## Abstract

**Background:**

Reactive oxygen species (ROS), including free radicals, oxygen ions, and peroxides, are implicated in cell damage. The objective of this study was to investigate whether the spontaneous production of ROS from neutrophils changes with age and is associated with the conventional inflammatory markers.

**Results:**

Thirty-seven elderly subjects (median age, 87, range 70–95 years) and 22 young subjects (median age, 26, range 21–37 years) participated in this study. Circulating levels of C-reactive protein, serum amyloid A, tumor necrosis factor-α, interleukin (IL)-1, IL-6, IL-8, monocyte chemotactic protein-1, and heat shock protein (HSP)70 were measured with enzyme-linked immunosorbent assays. The N-formyl-methionyl-leucyl-phenylalanine and lipopolysaccharide-stimulated ROS of neutrophils were quantified by flow cytometry. Both spontaneous ROS production and circulating levels of inflammatory markers were higher in the elderly group than in the younger group. In addition, spontaneous ROS production by neutrophils was negatively associated with HSP70 in plasma. We could not find the association between spontaneous ROS production by neutrophils and the other inflammatory markers including cytokines.

**Conclusion:**

The results suggest that spontaneous ROS production from neutrophils may increase with age and represent the different aspect of age-associated immune dysregulation.

## Background

Accumulating investigations have proven that elevated levels of several inflammatory mediators among apparently healthy men and women have predictive value for future adverse events [1-3]. In particular, prospective epidemiological studies have found increased vascular risk to be associated with increased basal levels of cytokines such as interleukin (IL) -6 and tumor necrosis factor (TNF)-α [4,5], and downstream acute-phase reactants such as C-reactive protein (CRP), fibrinogen, and serum amyloid A (SAA) [6,7]. The local inflammatory response is accompanied by a systemic response known as the acute-phase response [8]. The levels of those inflammatory markers increase several fold and decrease when the infection or trauma has healed. In contrast, chronic low-grade inflammation is typically characterized by mild to moderate increases in the systemic concentrations of these inflammatory markers [9]. Ageing is also associated with low-grade inflammation, and increases in circulating levels of CRP, TNF-α, and IL-6 have been identified in age-related inflammatory diseases in cross-sectional studies [10,11]. In this regards, circulating levels of these inflammatory mediators in patients and/or elderly humans may provide clinically useful information about the state of chronic medical disorders [12].

Neutrophils have a variety of functions including chemotaxis, adhesion to the endothelium and foreign agents, phagocytosis, and microbicidal activity. Neutrophils can penetrate and migrate into infected tissues, where they internalize and destroy invading microorganisms by producing toxic agents such as reactive oxygen species (ROS), proteases (elastase), and proteins that interfere with bacterial development (lactoferrin) [13]. ROS are generated during the complex process of respiratory burst, during which superoxide anion (O_2_^-^) is formed immediately after the reduction of molecular oxygen by single electrons through the nicotinamide adenine dinucleotide phosphate (NADPH) oxidase system [13]. O_2_^- ^is then rapidly transformed through enzyme activity (superoxide dismutase, catalase, myeloperoxidase) into other ROS, which include hydrogen peroxide (H_2_O_2_), hydroxyl radicals, and hypochlorous acid [13]. The importance of O_2_^- ^and H_2_O_2 _production for neutrophil bactericidal activity is illustrated by patients with chronic granulomatous disease who do not produce enough O_2_^.- ^and H_2_O_2_, thus becoming more susceptible to bacterial infection [14]. Despite the intrinsic function of neutrophils in the innate immune response, potent cytotoxic compounds are released into the extracellular space, which damage host tissues [13]. Herman's free radical theory, which has been accepted as a plausible explanation of the primary chemical reactions involved in ageing process, proposes that oxygen-derived free radicals are responsible (due to their high reactivity) for the age-associated damage at the cellular and tissue levels through the oxidative modification of biological molecules (lipids, proteins, and nucleic acids), which leads to functional impairment [15]. Cells that use oxygen and consequently produce ROS have evolved complex antioxidant defense systems to neutralize ROS and protect themselves against free radical damage. The increased oxidative stress in ageing is likely to be the consequence of an imbalance between free radical production and antioxidant defenses (with a higher production of the former) [16]. From a physiological perspective, endogenous ROS produced by the NADPH oxidase system regulate tyrosine phosphorylation dependent pathways that in turn modulate host defense responses such as phagocytosis and, via nuclear factor-κB, the expression of cytokines and chemokines that further modulate the inflammatory response [17]. Under pathological circumstances such as inflammatory tissue injury, excess production of ROS may destroy vicinal cells such as endothelium or epithelium [18]. In addition, since ROS are membrane permeable, they may influence intracellular signaling pathways in the cell as well as the adjacent cells in the inflammatory milieu [19]. During a systemic inflammatory response, it is feasible that ROS act as signaling molecules leading to modulation of crucial events including phagocytosis, secretion, gene expression, and apoptosis, resulting in dysregulation of inflammation [19]. In this regards, we hypothesized that the spontaneous production of ROS from neutrophils might change with ageing and associated with the conventional inflammatory markers, even though the intrinsically microbicidal task of ROS decreases with ageing. To examine the hypothesis, we compared ROS production by neutrophils and the circulating levels of inflammatory markers in older and younger people and investigated whether the elevated ROS levels were associated with inflammatory markers including cytokines. The objective of this study was to determine whether spontaneous ROS production by neutrophils is an effective inflammatory marker.

## Results

### Subject characteristics

Demographic, clinical, and blood chemistry data are shown in Table 1. There were no significant differences between the aged and young subjects in body mass index, white blood cell (WBC) counts, lymphocyte counts, neutrophil counts, total bilirubin (T-BIL), total cholesterol (T-CHO), high-density lipoprotein cholesterol (HDL-CHO), low-density lipoprotein cholesterol (LDL), free fatty acid (FFA), or lipid peroxide between the aged subjects and the young subjects (Table 1). The albumin level (P < 0.01) was significantly lower in the aged subjects than in the young subjects, while the triglyceride (P < 0.05) and glucose levels (P < 0.01) were significantly higher compared with the young subjects.

**Table 1:** Subjects' characteristics

	Old n = 37				Young n = 22		
	Median	Minimum	Maximum		Median	Minimum	Maximum

Age (year)	87	70	95	**	26	21	37
Weight (kg)	46.8	26.5	85.9	**	59.3	43.0	80.0
BMI (kg/m2)	21.45	13.4	32.3		21.2	17.8	26.7
WBC (105 cells/ml)	46.0	33.0	73.0		53.0	29.0	94.0
Lymphocytes (105 cells/ml)	18.5	7.8	27.9		18.2	10.4	40.0
Neutrophils (105 cells/ml)	22.5	12.3	50.2		29.4	12.6	68.0
Albumin (g/l)	42.0	35.0	46.0	**	46.0	42.0	51.0
Total billilbin (mg/l)	5.0	2.0	10.0		5.0	3.0	12.0
Total cholesterol (g/l)	1.95	1.3	2.68		1.85	1.29	2.50
High density lipoprotein (g/l)	0.56	0.32	1.00		0.62	0.34	0.87
Low density lipoprotein (g/l)	1.12	0.64	1.90		0.98	0.50	1.52
Triglyceride (g/l)	0.8	0.45	3.06	*	0.58	0.40	2.99
Free fattty acid (mEq/l)	0.5	0.14	0.93		0.45	0.12	1.07
Lipid peroxide (nmol)	0.4	0.1	0.6		0.4	0.1	0.7
Glucose (g/l)	0.91	0.74	1.76	**	0.83	0.70	0.97

Data are presented as median and range Mann-Whitney U test *; P < 0.05, **; P < 0.01, statistically identified as significant differences between old and young group BMI; Body Mass Index, WBC; White Blood Cells

### Inflammatory markers and cytokines

Differences in the levels of inflammatory markers and cytokines between the aged and the young subjects are shown in Table 2. The IL-8 (P < 0.01), TNF-α (P < 0.01), IL-6 (P < 0.05), CRP (P < 0.05), and heat shock protein (HSP) 70 levels (P < 0.05) were all significantly higher in the aged group than in the younger group. However, there were no significant differences in monocyte chemotactic protein (MCP)-1 or SAA. The ratio of spontaneous ROS production and inflammatory markers between the aged and the young groups was as follows: spontaneous ROS production, 1.2; IL-8, 1.4; MCP-1, 0.9; TNF-α, 1.8; IL-6, 1.4; CRP, 2.0; SAA, 1.1; and HSP70, 2.1.

**Table 2:** Inflammatory markers, and cytokines

	Old n = 37				Young n = 22		
	
	Median	Minimum	Maximum		Median	Minimum	Maximum
IL-8 (pg/ml)	25.6	13.8	81.2	**	18.3	8.6	70.7
MCP-1 (pg/ml)	184.3	111.5	5828.0		200.4	126.1	340.7
TNF-α (pg/ml)	0.91	0.20	3.38	*	0.52	0.11	1.69
IL-6 (pg/ml)	1.16	0.48	12.68	*	0.84	0.24	2.31
CRP (mg/dl)	0.41	0.04	51.62	*	0.21	0.08	0.70
SAA (μg/ml)	19.9	8.4	159.3		18.4	9.0	142.3
HSP70 (pg/ml)	36.2	0.0	166.0	*	16.8	0.0	47.0

Data are presented as median and range Mann-Whitney U test *; P < 0.05, **; P < 0.01, statistically identified as significant differences between old and young group IL-8, Interluikin 8; MCP-1, monocyte chemotactic protein-1; TNF-α, tumor necrosis factor–α; CRP, C-reactive protein; SAA, serum amyloid A; HSP, heat shock protein

### Neutrophil activity and spontaneous ROS production

The spontaneous ROS production (no stimuli) levels in the aged subjects were significantly higher than those in the young subjects (P < 0.01) (Table 3). On the other hand, there were no significant differences in N-formyl-methionyl-leucyl-phenylalanine (fMLP)- or lipopolysaccharide (LPS)-stimulated ROS production or the median channel in ROS production (fMLP or LPS-stimulated ROS production minus spontaneous ROS production) between the aged and young subjects (Table 3).

**Table 3:** Comaprison of ROS between ages

	Old n = 37				Young n = 22		
	
	Median	Minimum	Maximum		Median	Minimum	Maximum
Spontaneous	85.4	47.4	302.3	**	67.3	28.0	107.5
fMLP stimulation	170.0	91.6	500.3		143.0	89.0	204.9
fMLP stimulated Median channel	490.3	271.3	612.4		492.2	214.4	545.3
LPS stimulation	179.4	86.8	495.8		149.9	103.4	251.2
LPS stimulated Median channel	503.5	302.1	601.3		504.7	231.2	577.7

Data are presented as median and range Mann-Whitney U test **; P < 0.01, statistically identified as significant differences between old and young group

### Correlations

Table 4 lists the correlations between inflammatory markers and neutrophil counts, the spontaneous ROS production, and fMLP- or LPS-stimulated ROS production. The neutrophil count was significantly correlated with SAA (r = 0.46, P < 0.01), IL-6 (r = 0.48, P < 0.01), TNF-α (r = 0.37, P < 0.05), and CRP (r = 0.44, P < 0.05) in the aged group and IL-8 (r = 0.70, P < 0.05) in the young group. HSP70 was negatively related to the spontaneous ROS production in the aged group (r = -0.41, P < 0.05). The median channel in fMLP-induced ROS production or LPS-induced ROS production were not associated with any inflammatory markers including cytokines in the aged and young groups.

**Table 4:** Correlation analysis between ROS production and inflammatory markers.

	Neutrophil counts		Spontaneous ROS production	
	
	Old	Young	Old	Young
IL-8	-0.22	0.70 *	0.00	0.44
MCP-1	-0.10	0.44	-0.28	0.14
TNF-α	0.37 *	0.02	0.29	0.49
IL-6	0.48 **	0.27	0.13	0.35
CRP	0.44 *	-0.07	0.13	0.36
SAA	0.47 **	-0.18	-0.13	0.22
HSP70	0.00	0.47	-0.41 *	0.12

Median channel of	fMLP-stimulated ROS production		LPS-stimulated ROS production	

	Old	Young	Old	Young

IL-8	0.36	0.00	-0.15	0.31
MCP-1	-0.18	0.16	0.00	0.41
TNF-α	0.36	0.11	-0.28	-0.04
IL-6	0.34	0.09	0.18	0.32
CRP	0.23	0.28	-0.08	0.16
SAA	0.29	0.49	-0.29	0.34
HSP70	-0.04	0.12	0.14	0.40

*; P < 0.05, **; P < 0.01, statistically significant correlation coefficient by Spearman.

## Discussion

Vulnerability to infection in the elderly may result from reduced neutrophil numbers at the site of infection, or an intrinsic reduction in bactericidal activity [20]. Nevertheless circulating neutrophil numbers are not altered with age [21]. Our result also showed that the neutrophil counts did not changed with age. The neutrophil counts are known as a predictor of the incidence of the low-grade inflammation-associated diseases like atherosclerosis [22,23]. Consistent with previous studies, our results have found that neutrophil counts in the aged group were weakly correlated with inflammatory markers, IL-6, SAA, TNF-α, and CRP (r = 0.48, 0.47, 0.37, and 0.44, respectively), and that the plasma concentration of the conventional inflammatory markers and cytokines including IL-8, TNF-α, IL-6, CRP, and HSP70 were higher in the aged group than in the younger group. In addition, there was a strong correlation between neutrophil counts and IL-8 in the young group. IL-8 is a potent neutrophil chemoattractants that gathers neutrophils, produces excess ROS, and may indicate the higher intrinsic function of neutrophils in the young group. On the other hand, we found that spontaneous ROS production by neutrophils was significantly higher in the aged group than in the young group (P < 0.01), showing that higher levels of spontaneous ROS production from neutrophils could increase with age. However, we did not find a correlation between spontaneous ROS production and any of the inflammatory markers except HSP70. Since the comparison of the magnitude of spontaneous ROS production and inflammatory markers between the aged and young groups was less than 2-fold, the lower magnitude of inflammatory markers and ROS production in this group may be insufficient to examine the low-grade inflammation.

The spontaneous ROS production negatively correlated with HSP70 (r = -0.41, P < 0.05) only in the aged group, but not in the young group. HSP70 is a very conservative family of cytoprotective proteins that are specifically induced in response to several environmental stresses at the cellular level including heat shock, cellular energy depletion, oxidative stress or inflammation. Although the role of HSP70 in the blood (i.e. extracellular HSPs) is yet not known, HSP70 (intracellular HSP70) plays an important role in cytoprotection by preventing abnormal folding of newly synthesized polypeptides, or by assisting in the repair of damaged proteins [24]. The elderly people seem to be characterized by a diminished basal expression of HSP70 and a blunted induction in response to different stress conditions [25]. Therefore, the negative correlation between spontaneous ROS production and HSP70 may indicate that the higher concentration of HSP70 in plasma protectively responds to the oxidative stress [26], which may cause lower levels of spontaneous ROS production, or vice versa.

The response of fMLP- or LPS-stimulated ROS production was similar in the two groups. In general, the priming action of fMLP and LPS on neutrophil activity is seen as the body providing a global defense to support a primary immune response against exposure to antigens. Age-related neutrophil alterations show impaired ROS production and phagocytosis with a subsequently decreased capability to destroy bacteria [20,21]. The results may be the limitation in ROS detection. To determine the level of ROS production by neutrophils, we used a flow cytometric assay with whole blood samples. Hydroethidine (HE) is one of the most commonly used fluorescent probes to measure intracellular oxidant production. Oxidation of HE by O_2_^- ^produces ethidium, which can emit red fluorescence after intercalation with cellular DNA [27]. In this study, the elevated baseline level, in part, cause the difficulties to detect the response to the stimulation between the aged and young groups. Further study is needed to improve this point.

The main limitation of this study is that it did not involve aged healthy subjects without any condition known to be associated with low-grade inflammation. More specifically, the young group consisted of healthy individuals, whereas most subjects in the aged group had one or more conditions that could be associated with low-grade inflammation (hypertension [n = 13], dyslipidemia [n = 12], and diabetes mellitus [n = 2], which might have influenced the level of ROS production.

## Conclusion

We compared the ROS production by neutrophils and the circulating levels of inflammatory markers in older people and younger people, and investigated whether the elevated ROS levels are associated with inflammatory markers including cytokines. The results suggest that the spontaneous ROS production from neutrophils may increase with age and represent the different aspect of age-associated immune dysregulation. Further investigations on biological significance of ROS from neutrophils in ageing process are warranted

## Methods

### Subject characteristics

The 22 young subjects consisted of 12 men (age range, 21–37 years) and 10 women (age range, 22–37 years), and the 37 aged subjects who live in a nursing home consisted of 5 men (age range, 76–86 years) and 32 women (age range, 70–95 years). All subjects underwent a medical examination and blood biochemistry analysis. They were also interviewed by a physician. Moreover, the subjects were examined for any signs of infection and inflammation. None of the young subjects had any underlying acute or chronic disease that would affect the immune system nor did they have any cancers or cardiac, liver, brain, or kidney diseases. On the other hand, 13 of the aged subjects used anti-hypertension drugs, 2 had diabetes mellitus, 13 had dyslipidemia, and 12 had prior bone fractures (some had more than one of these conditions). None had suffered from bone fracture within 3 years. All participants were informed of the purpose and risks of the study, and written informed consent was obtained from them. The Research Ethics Committee at Waseda University approved the study.

### Biochemical and hematological characteristics

At between 7:00 and 8:00 am after an overnight fast, blood was collected from a forearm vein into a sterile tube containing heparin (Termo, Tokyo, Japan). WBC, lymphocyte, and neutrophil cell counts were obtained with an automatic cell counter pocH-100i (Sysmex, Kobe, Japan). The collected blood was kept at room temperature for 30 min and then centrifuged for 10 min at 1000 × g to separate serum. Samples were stored at -20°C until analysis. The levels of albumin, T-BIL, T-CHO, HDL-CHO, LDL-CHO, TG, FFA, lipid peroxide, and glucose were determined at a hematology laboratory (BML Corporation, Tokyo, Japan),.

### Inflammatory markers and cytokine measurement

The serum concentrations of CRP, SAA (Diagnostic Systems Laboratories Inc., Webster, TX, USA), HSP70 (Stressgen Biotechnologies Co., Victoria, BC, Canada), TNF-α (R&D Systems Inc., Minneapolis, MN, USA), IL-6 (R&D Systems), IL-8 (Becton Dickinson), and MCP-1 (R&D Systems) were measured by using commercially available enzyme-linked immunosorbent assay (ELISA) kits, according to the manufacturers' instructions. The absorbance was measured spectrophotometrically with a microplate reader (VersaMax Molecular Devices, Sunnyvale, CA, USA). The plasma concentration of each marker was calculated by comparison to a standard curve established in the same measurement.

### ROS from neutrophils

HE (Polysciences Warrington, PA, USA) was dissolved in N, N-dimethylformamide (Sigma-Aldrich, St. Louis, Missouri, USA) at a concentration of 400 mM. Before the flow cytometry assay, the stock solution was further diluted with phosphate buffered saline (PBS, from Wako Pure Chemical, Tokyo, Japan) to a final concentration of 20 mM. fMLP and LPS (Sigma) were used to stimulate the cells. The respiratory burst activity was adopted the method of Rothe et al. [27] using a flow cytometric assay. In the pilot experiments, we have confirmed that not catalase but superoxide dismutases (SOD) inhibits ROS production measured by HE in isolated neutrophils, and that sodium azide, which inhibits myeloperoxidase and SOD, enhances HE responses similar to another superoxide anion detection system [28]. Our method is considered to specifically measure superoxide anion produced by neutrophils.

Heparinized whole blood (100 μl) was placed in separate polypropylene tubes used for the stimulated tests and reagent blank tests. The HE solution was added to the samples, and the same volume of PBS was added to the reagent blank tube. All tubes were loaded with HE at 37°C for 5 min. Either fMLP (final concentration; 10^-5 ^mM) or LPS (final concentration; 1 μg/ml) was then added to the stimulated tubes. After a 35-min incubation at 37°C, 1.5 ml of FACS lysing solution (Becton Dickinson, San Jose, CA, USA) was added to all tubes. The tubes were left at room temperature for 10 min and then centrifuged at 1000 × g for 10 min. The supernatant was discarded, and the cells were resuspended in PBS before being centrifuged again. The washing procedure was repeated twice. After the third centrifugation, the supernatant was discarded and replaced with 400 μl of PBS.

**Figure 1:**
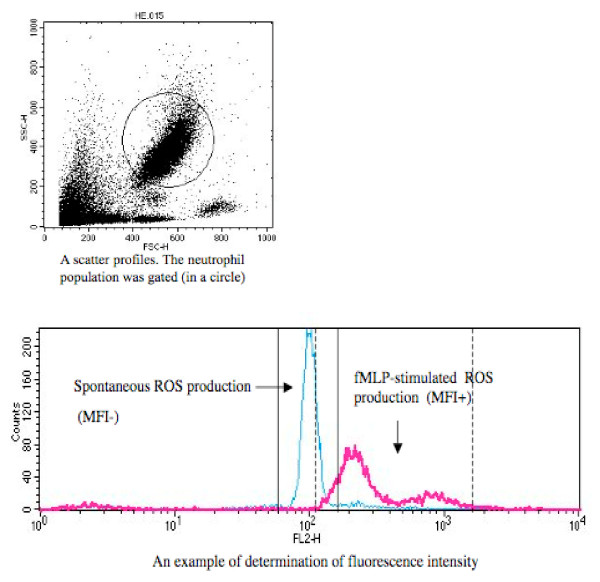
**An example showing a scattergram for gating the neutrophil population.** A scattergram of HE stained neutrophils (e.g. spontaneous ROS production) in the neutrophil-gated region, and the median channel of spontaneous ROS production (MFI-) and fMLP-stimulated ROS production (MFI+). The median channel for each assay was calculated from the following equation: median channel = 255.75 × log (MFI+ - MFI-), where MFI+ and MFI- are the median fluorescence intensities for a fMLP-stimulated neutrophil population and a no-stimulated neutrophil population, respectively.

All samples were then analyzed with a FACScan and CellQuest software (Becton Dickinson). Before acquiring data, the instrument was set up by using a reagent blank sample. The forward and side light scatter profiles were adjusted to ensure that the neutrophil population was clearly displayed. Fluorescence was measured on the FL2 red channel (wavelength, 480 nm), and the photomultiplier gain was adjusted so that the fluorescence of the reagent blank was confirmed to the first decade of the FL2 histogram display. Data were then collected from the reagent blank and stimulated sample tubes. A total of 10,000 events were collected for each sample. At analysis, the scattergram of forward and side scatter was displayed, and the neutrophil population was displayed by its location and selected by gating. A scattergram and a histogram of FL2 were then obtained for the neutrophil-gated region, and the median channel fluorescence was recorded from the display statistics. In order to make comparisons of fluorescence intensity between no-situmilation and stimulated ROS poroduction, we adopted the method of Shinkai et al. [29]; the median channel. The median fluorescence intensity (MFI) due to response in stimulation was calculated by subtracting the MFI for an unstimulated neutrophil population (MFI-) from the MFI on a stimulated neutrophil population (MFI+), the difference being expressed as a channel. Thus, the median channel for each assay was calculated from the following equation: median channel = 255.75 × log (MFI+ - MFI-). Fig. 1 shows an example.

### Statistical analysis

The statistical analysis was performed with SPSS 15.0J (SPSS Japan Inc. Tokyo, Japan). P values less than 0.05 were considered statistically significant. Data were presented as median, minimum and maximum. The Mann-Whitney U test was used to compare the values between the aged and the younger groups. Associations between parameters were evaluated using Spearman's correlation coefficient.

## Competing interests

The authors declare that they have no competing interests.

## Authors' contributions

KO had the overall responsibilities of the experiment design and statistical analysis, and wrote the manuscript. KS had the overall responsibilities of fund management and the experimental design. MO and KY conducted the experiments in the manuscript. SS had shared the concept and supported the manuscript. All the authors have read and approved the final manuscript.
